# Association between nasal airway minimal cross-sectional areas and obstructive sleep apnoea

**DOI:** 10.1093/ejo/cjad041

**Published:** 2023-08-08

**Authors:** Jessi Makkonen, Olli Tertti, Markus Rautiainen, Saara Markkanen, Olli Valtonen, Jaakko Ormiskangas, Ilkka Kivekäs, Timo Peltomäki

**Affiliations:** Department of Otorhinolaryngology—Head and Neck Surgery, Tampere University Hospital, Tampere, Finland; Faculty of Medicine and Health Technology, Tampere University, Tampere, Finland; Faculty of Medicine and Health Technology, Tampere University, Tampere, Finland; Department of Otorhinolaryngology—Head and Neck Surgery, Tampere University Hospital, Tampere, Finland; Faculty of Medicine and Health Technology, Tampere University, Tampere, Finland; Department of Otorhinolaryngology—Head and Neck Surgery, Tampere University Hospital, Tampere, Finland; Faculty of Medicine and Health Technology, Tampere University, Tampere, Finland; Department of Otorhinolaryngology—Head and Neck Surgery, Tampere University Hospital, Tampere, Finland; Faculty of Medicine and Health Technology, Tampere University, Tampere, Finland; Faculty of Medicine and Health Technology, Tampere University, Tampere, Finland; Faculty of Engineering and Natural Sciences, Automation Technology and Mechanical Engineering Unit, Tampere University, Tampere, Finland; Department of Otorhinolaryngology—Head and Neck Surgery, Tampere University Hospital, Tampere, Finland; Faculty of Medicine and Health Technology, Tampere University, Tampere, Finland; Faculty of Medicine and Health Technology, Tampere University, Tampere, Finland; Department of Oral and Maxillofacial Diseases, Tampere University Hospital, Tampere, Finland; Department of Oral and Maxillofacial Diseases, Kuopio University Hospital, Kuopio, Finland; Faculty of Health Sciences, Institute of Dentistry, University of Eastern Finland, Kuopio, Finland

**Keywords:** obstructive sleep apnoea, three-dimensional imaging, cone beam computed tomography, nasal cavity, cross-sectional area

## Abstract

**Background/Objectives:**

Patients with obstructive sleep apnoea (OSA) frequently present with some form of upper airway anatomical impairment. Considerable research has been conducted on the role of the structures of the jaw and pharynx in the pathogenesis of OSA; however, the significance of the nose is somewhat unclear. Computed tomography is a widely used imaging modality for assessing the nasal cavity and paranasal sinuses, but only a small amount of the acquired data is used. Our aim was to ascertain whether the size of the cross-sectional areas of the nasal airway, measured from cone beam computed tomography (CBCT) images, is associated with OSA severity.

**Materials/Methods:**

A total of 58 patients with OSA, without any major paranasal sinus inflammatory pathology, were included in this register-based study. Patients had previously undergone ambulatory polysomnography and CBCT. The cross-sectional areas of the nasal cavity were measured in CBCT coronal sections. Statistical analyses were performed to determine any correlation between the cross-sectional area measurements and apnoea–hypopnoea index (AHI) or any significant difference in cross-sectional areas between AHI severity groups.

**Results:**

No correlation was found between AHI and the smallest, total, or sum of the anterior cross-sectional areas of the nasal airway. Furthermore, there was no statistically significant difference in the cross-sectional areas between patients with the highest and lowest AHI.

**Conclusions/Implications:**

The small cross-sectional area of the anterior nasal cavity in patients without any major nasal pathology does not appear to be associated with OSA severity.

## Introduction

The spectrum of sleep-disordered breathing ranges from habitual snoring, the mildest form, to obstructive sleep apnoea (OSA), the most severe form (1). In OSA, repetitive narrowing or collapse of the upper airway occurs, and breathing is prevented for short periods during sleep.

In addition to overweight, most adult OSA patients have some impairment in the upper airway anatomy, such as retrognathic mandible, large tongue volume, narrow and long upper airway, and narrow upper dental arch/maxilla, which predisposes to the collapsibility of the upper airway (2–4). Furthermore, many OSA patients have non-anatomical factors contributing to airway obstruction, such as a low respiratory arousal threshold, ineffective upper-airway dilator muscles, and unstable ventilatory control (2,5).

The upper airway anatomically extends from the nostrils down to the epiglottal level. Within this ‘tube’, retropalatal and retroglossal areas are considered typical sites of upper airway obstruction in adults (6). The standard treatment for OSA is continuous positive airway pressure (CPAP) therapy, which prevents airway collapse (7). However, many patients with OSA also suffer from nasal obstruction (7–9), which can lead to nasal CPAP intolerance (7,8). Although studies on the nasal pathology of OSA patients are scarce, these patients seem to have more septal deformity, conchae bullosa, or turbinate hypertrophy (10,11). Nasal pathology may cause nasal obstruction and increased nasal resistance, limiting airflow in the upper airway. If CPAP therapy is not tolerated due to nasal pathology or, for example, low respiratory arousal threshold often found in normal-weight OSA patients (12), a mandibular advancement device (MAD) is recommended as an alternative therapy and may even be the initial treatment option. CPAP and MAD therapies are not curative methods, but when used, prevent upper airway collapse.

Maxillomandibular surgical advancement has been found to be the most effective and even curative surgical therapy for OSA (13,14). Recent systematic reviews (15,16) have reported that surgically assisted rapid maxillary expansion (SARME) alone may reduce OSA signs, i.e. decrease in apnoea–hypopnoea index (AHI) and increase in oxygen saturation. However, no association has been found between the amount of expansion and improvement in AHI (17). SARME is known to lead to an increase in the skeletal and dental transversal dimension of the maxilla (18,19), and an increase in volume of the nasal cavity and nasopharynx (20). These findings may indicate that nasal dimensions are related to the OSA severity. However, the role of the nose in the pathogenesis of OSA remains unclear.

Examination of the nasal airway cross-sectional area by acoustic rhinometry has suggested that the area in OSA patients may be smaller than in healthy controls (21). There has, however, been little previous research on the nasal airway volume of OSA patients using three-dimensional (3D) imaging. Rodrigues *et al*. (11) and Kim *et al*. (22) evaluated the nasal airway volume from computed tomography (CT) scans of OSA patients and found no difference in airway volume in comparison with healthy controls. In the nose, a narrow area can form an obstruction that limits airflow, even if the nasal cavity is wide elsewhere. Thus, volume is an insufficient value to assess the nasal airway since it does not acknowledge the narrow areas. The nasal valve area, which is located in the anterior part of the nasal cavity, is the narrowest portion of the nasal passage (23).

The aim of this study was to study the possible association between the narrowness of the nasal cavity and the severity of OSA in real-life patients. To achieve this, the cross-sectional areas of the nasal airway of adult OSA patients were measured from cone beam computed tomography (CBCT) scans and compared with polysomnography findings.

## Materials and methods

### Study design

**Figure 1:**
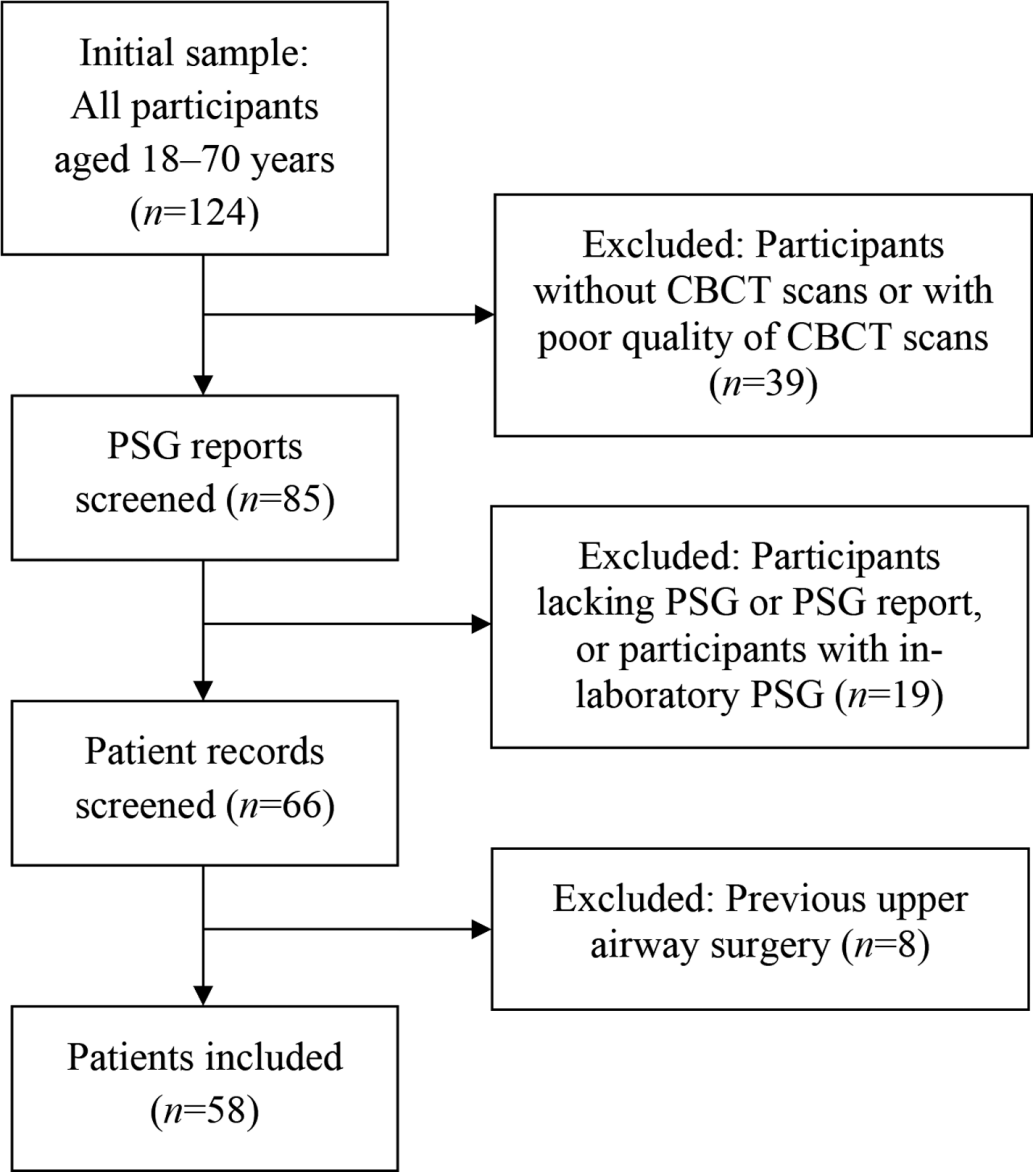
Flowchart illustrating the sample selection process.

This retrospective register-based study was approved by the Chief Physician of Pirkanmaa Hospital District. Ethics approval was not required. The study population consisted of patients with OSA referred for MAD therapy at the Department of Otorhinolaryngology and Oral Diseases at Tampere University Hospital. As part of the standard protocol for evaluating such patients, CBCT scans were performed to diagnose possible dental periapical pathologies, assess the airway and surrounding structures, and determine the optimal treatment plan. Due to the lack of prior studies with similar objectives, a formal a priori sample size calculation was not conducted. Instead, the sample size was determined based on the availability of eligible participants within the study period and the resources at our disposal. In total, 58 sequential patients were selected from the referral list. Patient data were gathered from the electronic medical records of Tampere University Hospital. The inclusion criteria for the study were patients aged 18–70 years who had been diagnosed with OSA based on ambulatory polysomnography (PSG). In addition, the patients had undergone maxillofacial and paranasal CBCT at Tampere University Hospital. The exclusion criteria were central sleep apnoea, poor-quality CBCT images, missing PSG data or in-laboratory PSG, and a history of upper airway surgery (Figure 1). In addition, basic patient characteristics, such as age, gender, height, weight, and body mass index (BMI), were gathered.

Half (52%) of the included patients were male, and the mean age was 47 years. Most of the patients were overweight (BMI 25–30) (48%) or obese (BMI > 30) (38%). According to the AHI, the severity of OSA was generally mild (50%) or moderate (38%) (Table 1). Most of the patients had tried CPAP therapy but failed CPAP compliance.

**Table 1.** Patient demographics.

	*n*	Min	Max	Mean	Median	*SD*
Sex	58					
Male	30 (52%)					
Female	28 (48%)					
Age (years)	58	22	67	47.6	49.0	12.0
BMI (kg/m^2^)	58	19.5	37.4	28.6	28.8	3.9
Normal (< 25)	8 (14%)					
Overweight (25–30)	28 (48%)					
Obese (>30)	22 (38%)					
AHI	58	5.0	58.0	16.7	14.9	10.9
Mild (5–15)	29 (50%)					
Moderate (15–30)	22 (38%)					
Severe (>30)	7 (12%)					
Cross-sectional areas (mm^2^)	58					
Smallest cross-section		36	302	166	164	45
Total		216	608	373	372	83
Anterior mean		328	825	470	452	89

BMI = body mass index; AHI = apnoea–hypopnoea index, SD = standard deviation. Cross-sectional areas are presented as follows: smallest cross-sectional area per single nasal cavity, total area representing the combined value of the smallest cross-sectional areas from both the left and right nasal cavities, and the anterior mean representing the combined mean area of the four anterior measuring points from both the left and right nasal cavities.

### Polysomnography

Patients had undergone ambulatory PSG at either Tampere University Hospital or in primary health care between 2014 and 2020. The PSG machine types most commonly used in this study were the Nox T3 system (Nox Medical Global, Reykjavik, Iceland) and Embla system (Natus Medical Incorporated, Middleton, WI, USA). Ambulatory PSG equipment consists of sensors that register nasal airflow, snoring, respiratory movements, total sleep time, sleep position, and pulse oximetry. The sleep study was conducted at home. AHI represents the number of apnoea or hypopnoea events per hour. Normal AHI is considered <5, and in cases of OSA the disease is classified as mild (AHI ≥ 5 and <15), moderate (AHI ≥ 15 and <30), or severe (AHI ≥ 30) (24).

### CBCT

All patients had undergone upright CBCT at the Department of Cranio and Dentomaxillofacial Radiology, Tampere University Hospital, between 2019 and 2020. The CBCT scans were acquired using Viso G7 (Planmeca, Helsinki, Finland) and Scanora 3Dx (Soredex, Tuusula, Finland). The CBCT scans were evaluated by one investigator using the Zinreich’s modified Lund–Mackay (LM) radiological staging system to assess for the possibility of chronic rhinosinusitis (25). This scoring system assesses each paranasal sinus, including the maxillary, frontal, sphenoid, anterior ethmoid, and posterior ethmoid sinuses, on a scale of 0–5 based on the extent of mucosal inflammation observed on the scan (0 = 0% inflammation, 1 = 1–25% inflammation, 2 = 26–50% inflammation, 3 = 51–75% inflammation, 4 = 76–99% inflammation, and 5 = 100% inflammation). The scores for each sinus on the right and left sides are then added together to give a total score that ranges from 0 to 50. In the present study population, the range of Zinreich’s modified LM staging scores was 0–15, with a mean value was 3.5. No nasal polyps were identified in any of the CBCT scans, indicating that none of the patients had any significant inflammatory pathology of the paranasal sinuses.

### Cross-sectional measurements of nasal airway

**Figure 2:**
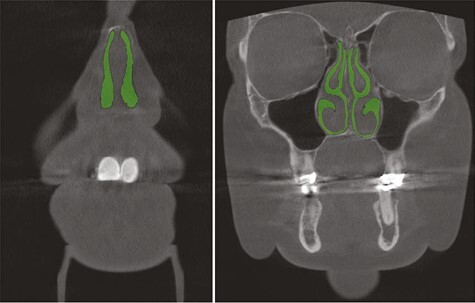
The cross-sectional area of the nasal cavity airway for the measurements marked in the image.

**Figure 3:**
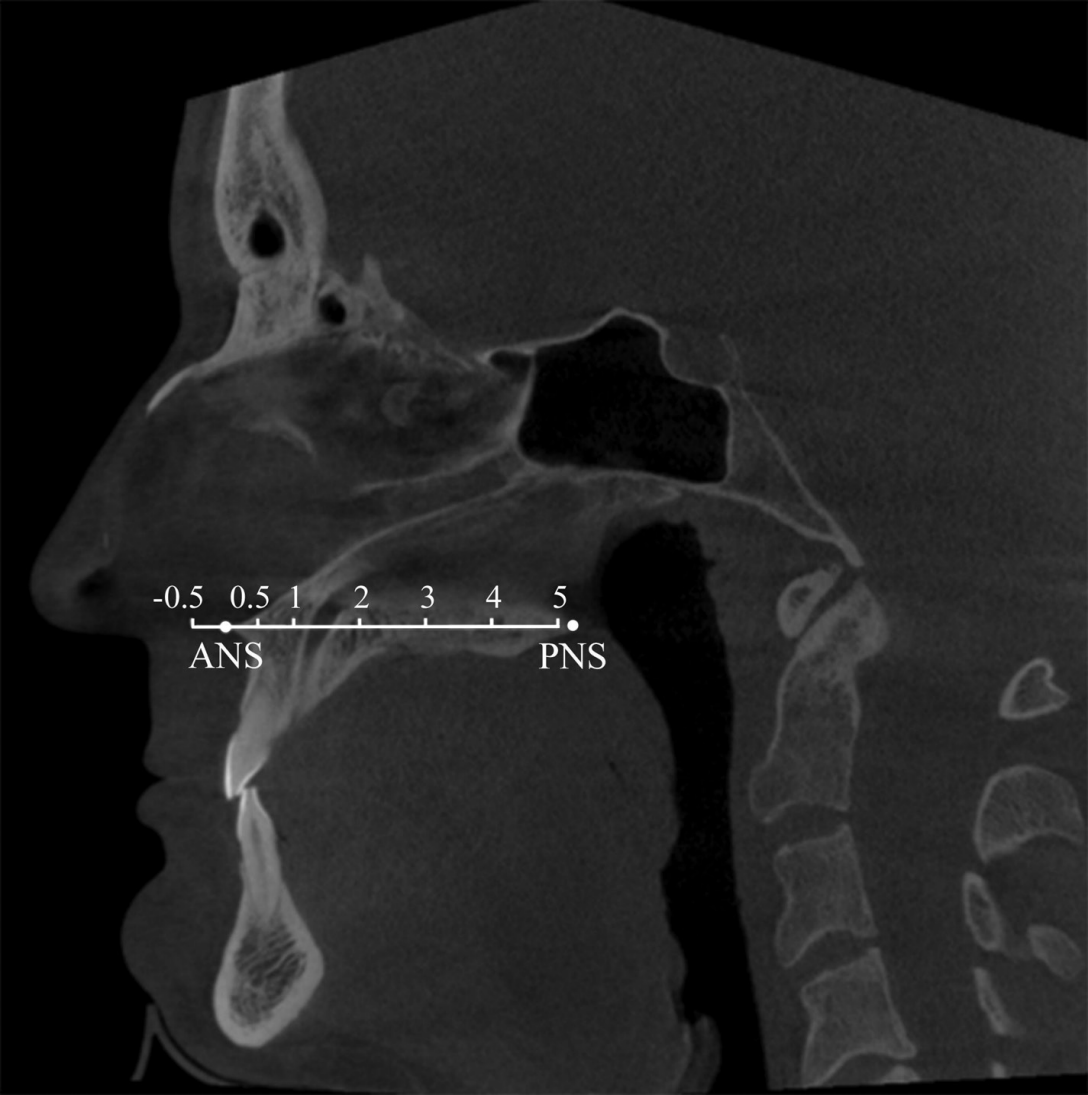
ANS = Anterior nasal spine of maxilla, PNS = posterior nasal spine of maxilla. Numbers above the line represent the distance (cm) from the ANS in which the coronal cross-sectional areas were measured.

One investigator performed all cross-sectional area measurements using 3D Slicer (version 4, Slicer Community, https://www.slicer.org/), a free and open-source software for medical image 3D analysis for research purposes (26). In order to minimize method error, another investigator performed single check-ups to ensure the accuracy of the measurements. The patient CBCT data were uploaded to the program and an analysis of the nasal cavity airway was performed. The cross-sectional areas of the free nasal airway in CBCT coronal sections were manually measured in square millimetres using the level tracing, paint, and erase tools of the Segment Editor tool (Figure 2). The measuring points used were the point of anterior nasal spine of maxilla (ANS), 0.5 cm anteriorly to ANS, 0.5 cm, 1 cm, 2 cm, 3 cm, 4 cm, and 5 cm posteriorly to ANS (Figure 3). The nasopharynx of some patients was achieved at 6 cm posteriorly to ANS. The right and left sides were measured separately and the smallest cross-sectional area of the nasal cavity from each side was identified from the coronal sections described above. Then, both minimum areas of each patient were added up and created the total minimum cross-sectional area to depict the narrowness of the nasal airway, since nasal breathing occurs on both sides simultaneously. The anterior part of the nose was then examined more accurately, and the mean cross-sectional area of the nasal airway of the first four anterior measuring points (between −0.5 and 1 cm) was explored, and the right and left mean areas were added together. These measurements were tabulated and compared to the AHI. Thereafter, 24 patients with the lowest (*n* = 12) and the highest (*n* = 12) AHI were divided into two groups to ascertain whether there was any difference in the previously described cross-sectional areas between the two groups.

### Statistical analysis

All data were analysed with SPSS Statistics (version 27 for Macintosh, IBM, Armonk, NY, USA). Wilcoxon test, Mann–Whitney U test, and Kruskal–Wallis tests were used to evaluate significant differences between variables. Since AHI data did not show a normal distribution, correlations between variables were evaluated with Spearman’s correlation test. *P* value < 0.05 was considered statistically significant.

## Results

The smallest single cross-sectional area varied from 36 to 302 mm^2^ (Table 1). In 91% (*n* = 106) of the nasal cavities (*n* = 116), the smallest cross-sectional area was in the first 2 cm anteriorly.

No statistically significant differences were found in the total cross-sectional areas (sum of the smallest cross-sectional areas of both nasal cavities) between genders (*P* = 0.16), AHI severity groups (*P* = 0.07), or BMI severity groups (*P* = 0.65). Furthermore, no correlation was found between AHI and the total cross-sectional areas (*P* = 0.07) (Table 2).

**Table 2.** Univariate Spearman correlations between AHI and cross-sectional areas.

	AHI	*P* value
	*R*	
Cross-sectional areas (mm^2^)		
Smallest cross-section	0.23	0.085
Total	0.24	0.073
Anterior mean	0.28 (0.01–0.5)*	<0.05
BMI	0.27 (0.002–0.5)*	<0.05

Cross-sectional areas are presented as follows: smallest cross-sectional area per single nasal cavity, total area representing the combined value of the smallest cross-sectional areas from both the left and right nasal cavities, and the anterior mean representing the combined mean area of the four anterior measuring points from both the left and right nasal cavities. R = correlation coefficient, AHI = apnoea–hypopnoea index, BMI = body mass index.

^*^95% confidence interval.

No statistically significant difference was found in the sum of mean anterior cross-sectional areas between genders (*P* = 0.06), AHI severity groups (*P* = 0.12), or BMI severity groups (*P* = 0.75). A statistically significant positive correlation (*r* = 0.28, *P* < 0.05) was, however, found between AHI and the mean anterior cross-sectional area (Table 2). There was no statistically significant difference in total cross-sectional areas between patients with the highest or the lowest AHI (*P* = 0.33) (Table 3). BMI showed no correlation with any cross-sectional areas.

**Table 3.** Comparison between groups.

	Group 1	Group 2	*P* value
AHI range			
Min	5.0	24.0	
Max	6.2	58.0	
Median	6.0	32.1	
SD	0.4	9.9	
Cross-sectional areas (mm^2^), median			
Smallest cross-section	153	173	0.33
Total	337	392	0.33
Anterior mean	437	463	0.12

Group 1: 12 patients with the lowest AHI; group 2: 12 patients with the highest AHI. Cross-sectional areas are presented as follows: smallest cross-sectional area per single nasal cavity, total area representing the combined value of the smallest cross-sectional areas from both the left and right nasal cavities, and the anterior mean representing the combined mean area of the four anterior measuring points from both the left and right nasal cavities. AHI = apnoea–hypopnoea index, SD = standard deviation.

## Discussion

The aim of the present study was to examine the association between the narrowness of the nasal cavity and the severity of OSA, with a particular focus on the anterior portion of the nose where the nasal valve area, which is the narrowest region of the nasal cavity, is located, and airflow restriction commonly occurs (23). Unexpectedly, patients with larger mean areas of the anterior nasal airway showed higher AHI, and there was a statistically significant positive correlation between AHI and mean anterior areas. This indicates that the narrowness of the nose is insignificant in the pathogenesis of OSA. Furthermore, we found no consistent association between AHI and the size of the nasal airway. Nevertheless, since the patients had a normal mucosal inflammatory status in the nasal cavity, the results may have been different if there had been significant nasal pathology.

Rodrigues *et al*. (11) studied the nasal airway volume of 91 adult subjects (71 with OSA and 20 healthy controls) with OSA varying from mild to severe. CT images in the supine position were acquired with a coronal slice thickness of 0.25 mm and an increment of 4 mm. The cross-sectional area of the nasal airway was measured from each slice, and the volume of the nasal cavity was estimated by computer. No significant difference in nasal airway volume between patients with OSA and healthy controls was found. Furthermore, they performed nasal endoscopy to evaluate nasal septum deviation and inferior turbinate hypertrophy. Nevertheless, no association was found between nasal airway volume and nasal pathology. Kim *et al*. (22) also evaluated the association between nasal airway volume and OSA including 109 patients. Of these, 91 had OSA with severity ranging from mild to severe, and 18 healthy controls. CT scans were acquired in a supine position with a slice thickness of 1 mm and an increment of 1 mm between slices. A computer was used to perform 3D segmentation and to estimate the volume of the nasal airway, including the maxillary sinuses. They found that the decreased maxillary sinus volume ratio to the total nasal airway volume was associated with the presence of OSA, but total nasal airway volume alone was not. However, the cross-sectional areas of the nasal airway were not measured.

Both Rodrigues *et al*. and Kim *et al*. (11,22) considered the association between nasal airway volume and OSA without defining the narrowness of the nasal cavity, even though most inspiratory resistance is caused in the anterior part of the nasal cavity (27). The narrow (or obstructed) area limits the nasal airflow, even when the nasal cavity is capacious in other areas. Thus, nasal airway volume alone is an insufficient variable to evaluate the effect of the nose on OSA pathophysiology. In the present study, we found no evidence that narrowness of the nasal airway predisposes to OSA. This finding is in line with previous research suggesting that the role of the nose in the pathogenesis of OSA is not crucial. Moreover, this finding also supports the prevalent clinical consensus that the treatment of nasal obstruction does not treat OSA (8,28). However, normal nasal breathing is essential for the success of CPAP and MAD therapy (29,30).

In previous studies on CBCT imaging of the upper airway of OSA patients, the focus has frequently been on the pharyngeal airway. Momany *et al*. (31) examined the CBCT scans of 22 patients with OSA and 23 healthy controls and searched for the narrowest area of the pharyngeal airway. When compared to healthy controls, patients with OSA were found to have a narrower airway. In previous studies, however, the minimal nasal cross-sectional area of OSA patients has only been studied using acoustic rhinometry. Moxness *et al*. (21) compared minimal cross-sectional areas between patients with OSA and healthy controls. In their study, 185 adults (93 patients with OSA and 92 healthy controls) without considerable nasal pathology were included. Acoustic rhinometry was performed before and after decongestion of the nasal mucosa. They found significantly smaller cross-sectional areas of the nasal airway in patients with OSA before and after decongestion, particularly in the anterior part of the nose. Our findings are not compatible with these findings, even though previous research has shown a correlation between acoustic rhinometry measurements and 3D volumetric measurements obtained from CBCT (32). In the present study, we found defining the cross-sectional areas of CBCT scans was more reliable, since acoustic rhinometry measurements are known to be only accurate in the anterior nasal cavity (33). CBCT offers a potential advantage in that it allows for the acquisition of multiple forms of information through a single research method, including measurements of the entire nasal cavity and any associated anatomical abnormalities that may contribute to nasal constriction. This can provide a more comprehensive understanding of the nasal airway and its contribution to OSA. In addition to improving the comprehensiveness of the analysis, CBCT may reduce the number of required studies, thus saving time and money. Moreover, CBCT has a low radiation burden, which enhances its safety profile (34).

Present findings suggest that there is no significant association between the severity of OSA and cross-sectional areas of the nose. However, this does not eliminate the possibility that SARME may have a positive impact on OSA signs and symptoms at an individual level, albeit indirectly, without any increase in nasal cavity volume. Two possible explanations have been proposed, both of which are believed to be secondary effects of maxillary expansion. Firstly, SARME is recommended for patients with maxillary transverse deficiency, often accompanied by a lateral crossbite. Expansion of the maxilla/dental arch has been suggested to lead to a change in the tongue posture, which results in enlargement of the pharyngeal airway at the sites of critical obstruction (35,36). Secondly, an increase in nasal volume, particularly at the constriction site, has been shown to decrease nasal airflow velocity and reduce the negative pressure of the airway during inspiration while sleeping (17).

## Strengths and limitations

A strength of our study was the study population which consisted of real-life patients with OSA without considerable nasal mucosal pathology. To our best knowledge, no previous studies have defined the narrowness of the nasal airway of patients with OSA using CT imaging. We acknowledge the limitations of the current study. The study population was limited, and the study was a retrospective register-based study. Due to the absence of previous studies with similar objectives, a formal a priori sample size calculation was not performed. We acknowledge that this may impact the statistical power and generalisability of our findings. Additionally, in our study, polysomnography was carried out with the patients in a supine position while the CBCT scans were taken with the patients in an upright position. It is important to note that changes in posture can affect airway dimensions in the nose (37). The use of CBCT in an upright position allowed for a more realistic evaluation of the upper airway during wakefulness, which can differ from the airway dimensions observed during sleep. Future studies that evaluate airway dimensions in both supine and upright positions could provide a more comprehensive understanding of the upper airway and its contribution to OSA.

One aspect that can be criticised in our research is the feasibility of measuring the cross-sectional area at every individual slice of the CBCT. However, clinically significant anatomical structures that impact nasal airflow are not confined to just one or two slices of the nasal cavity; rather, they span across multiple slices. Therefore, measuring the cross-sectional area at each individual slice would be unlikely to provide additional clinically significant information.

Another aspect to consider in our study is the non-standardised nature of CBCT scan orientation. To address this limitation, we ensured that all CBCT scans were conducted at the same hospital using consistent equipment and following a standardised protocol. Additionally, we carefully reviewed all scans to ensure accurate identification of the nasal airway borders and excluded any scans with poor image quality or positioning that may have affected the measurements.

Furthermore, the study sample consisted of a relatively small number of severe OSA patients (*n* = 7). This may limit the generalisability of the findings to patients with severe OSA and restrict the analysis of risk factors associated with OSA severity. Therefore, further studies with larger samples of severe OSA patients are necessary to validate our findings. Nevertheless, despite the limited number of severe OSA patients, this study provides a valuable starting point for further investigation into the risk factors and treatment options for OSA.

In this study, the measurements used are static radiological measurements, but nasal airflow dynamics is a far more complex matter. The information CT provides is vast, but only a limited amount of this information is used for clinical purposes. Therefore, further research on 3D imaging and computational fluid dynamics is needed to improve the diagnostics of OSA. In addition, future research on the role of the nose in the pathogenesis of OSA is also required. The present study contributes to the understanding of the complex pathophysiology of OSA and highlights the importance of a multidisciplinary approach to its diagnosis and management. Future research should focus on identifying novel treatment strategies that target different anatomical and physiological factors involved in OSA development and progression.

## Data Availability

All data are available upon request.
